# Perspective: plasticity, the enemy of the good

**DOI:** 10.20517/cdr.2019.23

**Published:** 2019-06-19

**Authors:** Sarah Yvonnet, Anouk Barberousse, Alexandre E. Escargueil

**Affiliations:** ^1^Sorbonne Université, CNRS, Laboratory Sciences, Normes, Démocratie (SND), Paris F-75005, France.; ^2^Sorbonne Université, INSERM, Laboratory Cancer Biology and Therapeutics, Centre de Recherche Saint-Antoine, CRSA, Paris F-75012, France.

**Keywords:** Resistance, plasticity, metastasis, timeline, clinical action

## Abstract

Plasticity is an important feature of modern cancer research. However, the level at which we should consider it remains an open question. Such debate is not new in the field of cancer and can be exemplified by the different models explaining carcinogenesis. Those models mostly explain cell transformation through the deregulation of the internal circuitry. In the last years, those models dramatically increased our knowledge and led to a series of short-term successes in terms of therapeutics. However, cancer drug resistance inevitably arises. Recently, studies on the so-called tumor microenvironment enriched the cell-centered perspective but it also enlarged the complexity of cancer etiology in particular for advanced diseases. Here, we suggest that the plastic and multi-sites specific nature of cancer combined with our incapacity to promise cure should push towards a new perspective where early clinical actions, instead of late ones, should be heralded as the priority of cancer research and care.

Plasticity is becoming a key feature of Cancer research and its understanding is raising new challenges for caregivers and decision makers^[1]^. However, the level to which we shall consider plasticity (e.g., tumor or cancer cell levels) remains a questionable issue that might yet have important consequences in terms of drug development, cancer patients’ care and future medical interventions. Such a debate is not particularly new. Cancer research is indeed a field particularly prone to the existence of different models. This might be exemplified with two models made to explain the etiology of the disease: the “multigenic and multiphasic model of cancer” and the “epigenetic progenitor model of cancer”^[2,3]^. The former one relies on the mutational activation of oncogenes and inactivation of tumor suppressor genes. In that model, oncogenes and tumor suppressor genes are particularly important because they can be thought as highly connected nodes in the genetic network capable of integrating signals controlling cell cycle and cell death^[4,5]^. Tumor formation then requires the accumulation of additional mutations determining the biological features of the tumor^[1]^. In the “epigenetic progenitor model of cancer”, it has been suggested that tumorigenesis first relies on an abnormality of the epigenetic network of progenitor cells that precedes mutations in tumor suppressor genes and oncogenes^[6]^. This is followed by a general epigenetic and genetic instability that is designing the evolution of the tumor mass^[6,7]^. Interestingly, both models share a global cell centered perspective [Figure 1]. Indeed, while the “multigenic and multiphasic model” describes the etiology of the disease through a succession of genes mutations^[4]^, the “epigenetic progenitor model” focuses on gene modulation events including modifications like gene hypomethylation or hypermethylation as the initial disruptive step leading to the development of the neoplasm^[6]^. Those two models however regard cancer as a disruption of the normal cell cycle and are grounded on the importance of the cell transformation through the dysregulation of its own internal mechanisms. Those two different models share so a common perspective that was described by Bertolaso as the “cell centered perspective”^[3]^.

**Figure 1:**
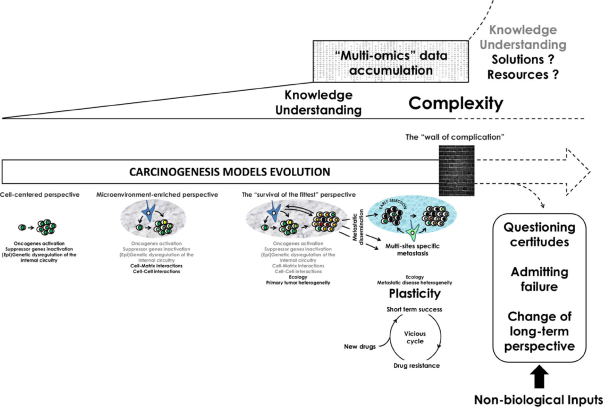
The increasing knowledge in the field of Cancer biology led to an outstanding improvement of our understanding of the pathology in the last 20 years but the recent and unprecedented accumulation of data makes it now very difficult to prioritize and stratify this overwhelming set of information. From an initial “cell-centered” perspective, rapidly enriched thanks to our comprehension of the role of the tumor microenvironment in cancer promotion and progression, we now reached a perspective in which ecological interactions between tumor cells and their surrounding environment design the multi-sites specific tumor landscape. Epigenetic and genetic heterogeneity of either primary or distant metastatic tumors is now the hallmark of advanced diseases in which cell and/or tumor plasticity become the rule. This makes development of new anticancer drugs shallow because of the constant emergence of resistance clones. In front of such a moving “wall of complication” and in a context of limited resources, the plasticity of the disease, and the complexity it generates, should challenge our certitudes and lead to a new perspective in terms of cancer research and care where biologists and non-biologists collaborate to critically assess scientific assumptions and to foster dialogue between sciences, as well as between science and society

The accumulation of complications: Each of the two models within the “cell-centered perspective” tends to identify new molecular and intracellular pathways and processes involved in cancer progression and the occurrence of new tumor cell capacities including those involved in drug resistance. This leads to an increased knowledge but also to an outstanding number of possible elements involved in the etiology of the pathology. So, while biologists collect still more data, it is now difficult to make a clear sense out of this overwhelming set of information. Already, in 2006, Hornberg *et al.*^[8]^ stated that: “Although knowledge of the molecular cell biology of cancer is enormous, at the same time, the emerging complexity of the entire ‘cancer system’ overwhelms us, leaving an enormous gap in our understanding and predictive power”. This “gap” between the accumulation of molecular knowledge and the understanding of the disease is now becoming problematic, because despite the enormous resources that are spent each year, cancer research did not provide a clear and actionable insight into the way the disease begins, progresses and reaches a highly plastic and heterogeneous structure in advanced diseases^[1,2]^. Therefore, it seems that the cell centered perspective is now confronted to what the philosopher Bertolaso called “The wall of complication” meaning that despite of the amount of informations, biologists are struggling to get a simpler and more comprehensive view of cancer^[3]^.

Time for a change? In front of such a “wall of complication” faced by the cell-centered perspective, biologists^[9,10]^ first proposed to look at the problem in a different way: “There is not solely a matter how to integrate all available knowledge in such a way that we can still deal with complexity, but we must be aware that a deeply transformation of the currently accepted paradigm is urgently needed”^[10]^. One of the justifications for such a change of perspective is that cancer cells are, by definition, not isolated. They are always in interactions with normal cells within the diseased tissue^[11]^. Tenants of a change proposed so that rather asking how normal cells can be transformed into cancerous cells, biologists should ask how multicellular tissues can be turned into tumors^[9,11]^. Here, cancer should be thought as the loss of tissue function and organization that is usually maintained by the interplay between parenchymal cells and their microenvironment^[11]^. In that context, the change of perspective mostly consists in addressing questions about carcinogenesis at a different biological level such as the potential role played by the microenvironment in cancer promotion and progression [Figure 1] and whether the microenvironment could be a therapeutic target in order to cure cancer^[1,9,11]^. However, while knowledge is still growing and data accumulating, demonstrating each day more complexity, we should now ask whether this is not the time for moving again to a new perspective. In metastatic disease, the microenvironment is indeed highly versatile and is capable to select for specific clones turning what was initially thought as a unique disease to a multi-sites specific disease^[12-16]^. In such a model, the “survival of the fittest” becomes the rule of cancer evolution and our promise to cure all cancer through new drugs development remains very unlikely and, in a way, shallow [Figure 1]. The “survival of the fittest” model, where cancer cells’ fitness mostly relies on their interactions with their surrounding environment, might so better define the process of tumorigenesis and its associated plasticity in terms of adaptive behaviors. Importantly, its conceptualization should also warn us on our real ability to act efficiently on such an “irrational” behavior of human cells. In other words, tumor plasticity might render our quest to cure cancer useless because cancer drug resistance will inevitably arise [Figure 1]. This vicious circle, made of short-term success and failure, can broadly be illustrated with the resistances that are developed in response to the use of tyrosine kinase inhibitors^[17-19]^. Thus, admitting our inability to treat such highly advanced diseases might help us to redirect the available resources to develop and clinically validate new strategies for either detecting early cancer where clinical actions are efficient or assessing the risk of recurrence of early diagnosed neoplasms^[20,21]^. In that perspective, the characterization of the precise timeline of cancer dissemination might help to focus researches and resources towards the detection of early disseminating neoplasms with poor clinical outcome^[22,23]^. The recent success obtained against Human Papilloma Virus-induced cervical cancer might, in a way, highlight the need to precisely characterize timelines of disease development and dissemination to prevent and control cancer^[24]^. However, to be entirely efficient, prophylactic and preventive actions against cancer need to be a call to action for the world health community to facilitate access to new preventive and screening technologies and to increase their acceptance by the global communities.

This change of perspective will certainly not solve all the unanswered questions regarding cancer. It also does not mean that a cell-centered perspective has to be dropped out, or that it does not work as it led to many successes^[25]^. However, it may contribute to make sense of “the vast body of essentially incoherent phenomena that constituted cancer research”^[2]^ and to focus cell centered researches to rare neoplasms where either early detection is not possible or treatment not, or poorly, accessible. Obviously, this change of perspective should be part of a long term action involving not only biologists or clinicians but also human, political and public health sciences. In such a perspective, non-biologist inputs will strikingly be required to foster dialogue between cancer biology, society and decision makers and to challenge certitudes [Figure 1]. Accordingly, in a recent article, Laplane and colleagues underline the need to re-implement philosophy in sciences where philosophy might contribute to “the clarification of scientific concepts, the critical assessment of scientific assumptions or methods, the formulation of new concepts and theories, and the fostering of dialogue between different sciences, as well as between science and society”^[26]^. The aim here is that, maybe, biologists have the opportunity to switch to a different perspective that could open up new complementary axes of research that hopefully would break the scheme of the short-term success before facing complications and cases of resistance^[3]^.

## Declarations

### Copyright

© The Author(s) 2019.
